# A bibliometric analysis of exertional heat stroke research in Web of Science

**DOI:** 10.1186/s40779-016-0101-6

**Published:** 2016-10-20

**Authors:** Zhi Mao, Chao Liu, Shuo Chen, Zheng-Guo Zhu, Hong-Jun Kang, Fei-Hu Zhou

**Affiliations:** 1 Department of Critical Care Medicine, Chinese PLA General Hospital, Beijing, 100853 China; 2 Department of Medical Information, Chinese PLA General Hospital, Beijing, 100853 China; 3 Department of Orthopedics, Chinese PLA General Hospital, Beijing, 100853 China

**Keywords:** Heat stroke, Bibliometric analysis

## Abstract

**Background:**

Exertional heat stroke is a fatal condition and remains a health problem. This paper evaluates the publication trend regarding exertional heat stroke research between 1996 and 2015 using a bibliometric method.

**Method:**

Articles regarding exertional heat stroke research published between 1996 and December 2015 were searched for in the SCI-EXPANDED database of Web of Science. The search results were analyzed with regard to publication year; publication quantity regarding countries/regions, and authors; citation frequency; and journal distribution. CiteSpace (v3.6) was used for a document co-citation visualization analysis.

**Results:**

In total, 289 publications on heat stroke were located. After selection, 209 original articles conducted across 28 countries/regions and published in 83 journals were included in the analysis. The USA, Isreal, and France were the most common locations for exertional heat stroke studies. The CiteSpace visualization cluster analysis showed that exertional heat stroke-related mortality and protective measures were constant concerns of research.

**Conclusions:**

Research related to exertional heat stroke has been continuous concerned. USA is still the leading country in this field.

## Background

Heat stroke is a severe and fatal condition clinically characterized by a severe rise in core body temperature (often >40 °C), with concomitant central nervous system dysfunctions such as delirium, convulsions, epilepsy, and coma [1]. Heat stroke is primarily classified into classic heat stroke and exertional heat stroke. The latter is particularly encountered during modern warfare and military training. Over the past few decades, the incidence of exertional heat stroke has significantly increased. According to data from the US Centers for Disease Control and Prevention, 7,000 cases of heat stroke death occurred in the US between 1979 and 1997 [2]. Along with the change in global climate, the progress of urbanization, and the extension of life expectancy, exertional heat stroke is expected to remain a health problem that cannot be ignored [3]. The most common treatment for exertional heat stroke is rapid cooling to promptly lower body temperature to normal. This strategy can reduce the risk of organ damage [4]. Despite effective cooling, numerous patients suffer from multiple organ failure, disability, and even death following active cooling treatment. Several studies related to exertional heat stroke have been published over the last few decades [1–3]. However, many problems remain to be addressed regarding the pathogenesis, prevention, and treatment of exertional heat stroke [3]. Bibliometric analysis is a wildly used method to evaluate the publication trend on a special topic [5].

Although many papers investigated exertional heat stroke, reports are still currently lacking regarding the trend of exertional heat stroke publications. We aimed to employ a bibliometric method to analyze the trend of exertional heat stroke publication in the latest twenty years.

## Methods

This trend analysis was performed using the Web of Science database with regard to publication quantity, country/region, institution, author, journal, and so on. The co-citation patterns were visualized to provide evidence for relevant clinics and research.

### Data source and retrieval

The SCI-EXPANDED database of Web of Science was searched, and the last search occurred on June 14, 2016. The search terms “heat stroke” or “heatstroke” and “exertional” were used to create the following search queries: (topic = “heat stroke”) OR (topic = heatstroke)) AND “exertional”. The time span was set to between 1996 and 2015. The publication type was not limited, and “article” was selected for an in-depth analysis.

### Statistical analyses

Histcite 12.03.07 (Thomson Reuters) was used for the descriptive analysis. A bibliometric method was used to quantitatively describe the published articles regarding year of publication; publication quantity, including country/region, institution, and author; citation frequency; and journal distribution. A citation map was generated. The co-citation visualization analysis was performed using CiteSpace 3.6.

## Results

### Selection of articles

Using the search queries, 289 publications were searched, including 209 original articles, 37 reviews, 11 editorials, 10 meeting abstracts, 10 letters, 8 proceedings papers, 4 corrections. Based on the selection criteria, 80 non-original articles were excluded, and 209 original articles related to exertional heat stroke were included in the analysis.

### Distribution of articles by publication years

The quantity of published articles on exertional heat stroke showed an overall trend by year, which rose from 12 in 1996 to 31 in 2015 (Table 1).

**Table 1:** Number and citation frequency of published articles on extertional heat stroke between 1996 and 2016

Publication year	Articles	Citation frequency	Average citation frequency
1996	12	485	40.4
1997	4	38	9.5
1998	6	105	17.5
1999	3	135	45.0
2000	1	24	24.0
2001	6	82	13.7
2002	6	93	15.5
2003	10	98	9.8
2004	11	318	28.9
2005	7	213	30.4
2006	12	244	20.3
2007	12	250	20.8
2008	7	117	16.7
2009	10	192	19.2
2010	11	151	13.7
2011	16	120	7.5
2012	9	111	12.3
2013	12	61	5.1
2014	23	43	1.9
2015	31	43	1.4
Total	209	2923	14.0

### Distribution of articles by countries and regions

The 209 articles originated from 28 countries and regions. The USA, Isreal, France, Mainland China and Taiwan, and UK were the most common locations for publishing articles on exertional heat stroke (Table 2). USA is the leading country during the twenty years in publishing articles on exertional heat stroke.

**Table 2:** An analysis of the number and citation frequency of published articles with regard to major countries/regions

Rank	Country/region	Total records	Publication year																				Total citation
			1996	1997	1998	1999	2000	2001	2002	2003	2004	2005	2006	2007	2008	2009	2010	2011	2012	2013	2014	2015	
1	USA	105	8	0	3	0	1	3	4	4	3	3	7	6	2	4	7	10	3	5	10	22	1725
2	Israel	19	0	0	0	1	0	0	1	2	6	0	2	0	0	0	0	4	1	1	1	0	266
3	France	14	1	1	0	1	0	1	0	0	0	1	1	0	1	1	2	0	0	0	2	2	177
4	Mainland China	13	0	0	0	0	0	0	0	0	0	0	0	1	0	0	0	2	1	1	4	4	37
5	UK	12	1	0	0	0	0	0	0	0	0	1	0	2	1	0	0	0	1	2	0	4	283
6	Taiwan	11	1	2	0	0	0	0	0	1	2	0	0	0	0	0	2	1	1	0	1	0	109

### Distribution of articles by authors

The 209 articles were written by 803 authors in total. The top 10 authors publishing articles on exertional heat stroke primarily came from the US and Israel (Table 3). Casa DJ from the University Connecticut of USA published the most articles (20 records) and accounted for 9.6 % of all published articles. Casa DJ published the first article on exertional heat stroke in 2005 [6]; the most recent research on exertional heat stroke was published in 2015 [7].

**Table 3:** Ten authors who published at least 10 articles on exertional heat stroke from 1996 to 2016

Rank	Author	Records	Citations	Average citation	Country/region	Institution
1	Casa DJ	20	302	15.1	USA	University Connecticut
2	Armstrong LE	12	325	27.1	USA	University Connecticut
3	Epstein Y	9	253	28.1	Israel	Tel Aviv University
4	Moran DS	9	203	22.6	Israel	Tel Aviv University
5	Roberts WO	8	241	30.1	USA	University of Minnesota
6	Wenger CB	8	255	31.9	USA	US Army Research Institute of Environmental Medicine
7	Heled Y	7	131	18.7	Israel	Heller Institute of Medical Research
8	Maresh CM	7	193	27.6	USA	University Connecticut
9	Gardner JW	6	191	31.8	USA	Uniformed Services University of the Health Sciences
10	McDermott BP	6	114	19.0	USA	University of Tennessee

### Distribution of articles by journals

The 209 articles were published across 105 journals. The top 10 journals published 83 articles on heat stroke and accounted for 32.9 % of all articles included in this study (Table 4). The *JOURNAL OF ATHLETIC TRAINING* published the most articles (16). Ellis A published the most frequently cited article in *Gut* in 1996 which was referenced 102 times [8].

**Table 4:** Top ten SCI journals for heat stroke publications

Journals	Records	Categories	Quartile	F2014
Journal of athletic training	16	Sport sciences	Q2	2.017
Medicine and science in sports and exercise	16	Sport sciences	Q2	3.983
Military medicine	12	Medicine, general & internal	Q3	0.911
Aviation space and environmental medicine	8	Medicine, general & internal/public, environmental & occupational health/sport sciences	Q3/Q4/Q5	0.875
Journal of applied physiology	7	Physiology/sport sciences	Q2/Q1	3.056
Wilderness & environmental medicine	6	Public, environmental & occupational health/sport sciences	Q3/Q3	1.196
European journal of applied physiology	5	Physiology/sport sciences	Q3/Q2	2.187
Journal of thermal biology	5	Biology/zoology	Q2/Q2	1.505
American family physician	4	Primary health care/medicine, general & internal	Q1/Q2	2.175
Journal of strength and conditioning research	4	Public, environmental & occupational health/sport sciences	Q3/Q3	1.196

### Citation map

**Fig. 1:**
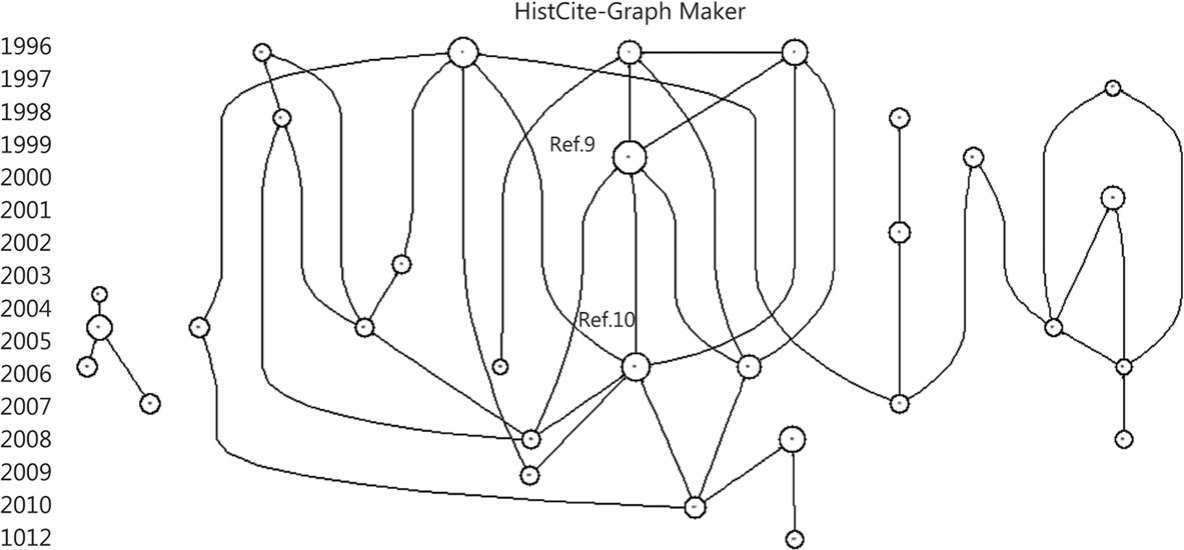
A citation map, with Ref 9 and Ref 10 as core documents

One of the core documents cited was published by Epstein Y in 1999, titled “Exertional heat stroke: a case series” [9]. Another core document cited was published by Smith JE in 2005, titled “Cooling methods used in the treatment of exertional heat illness” [10] (Fig. 1).

### Analysis of time-frequency of key words

**Fig. 2:**
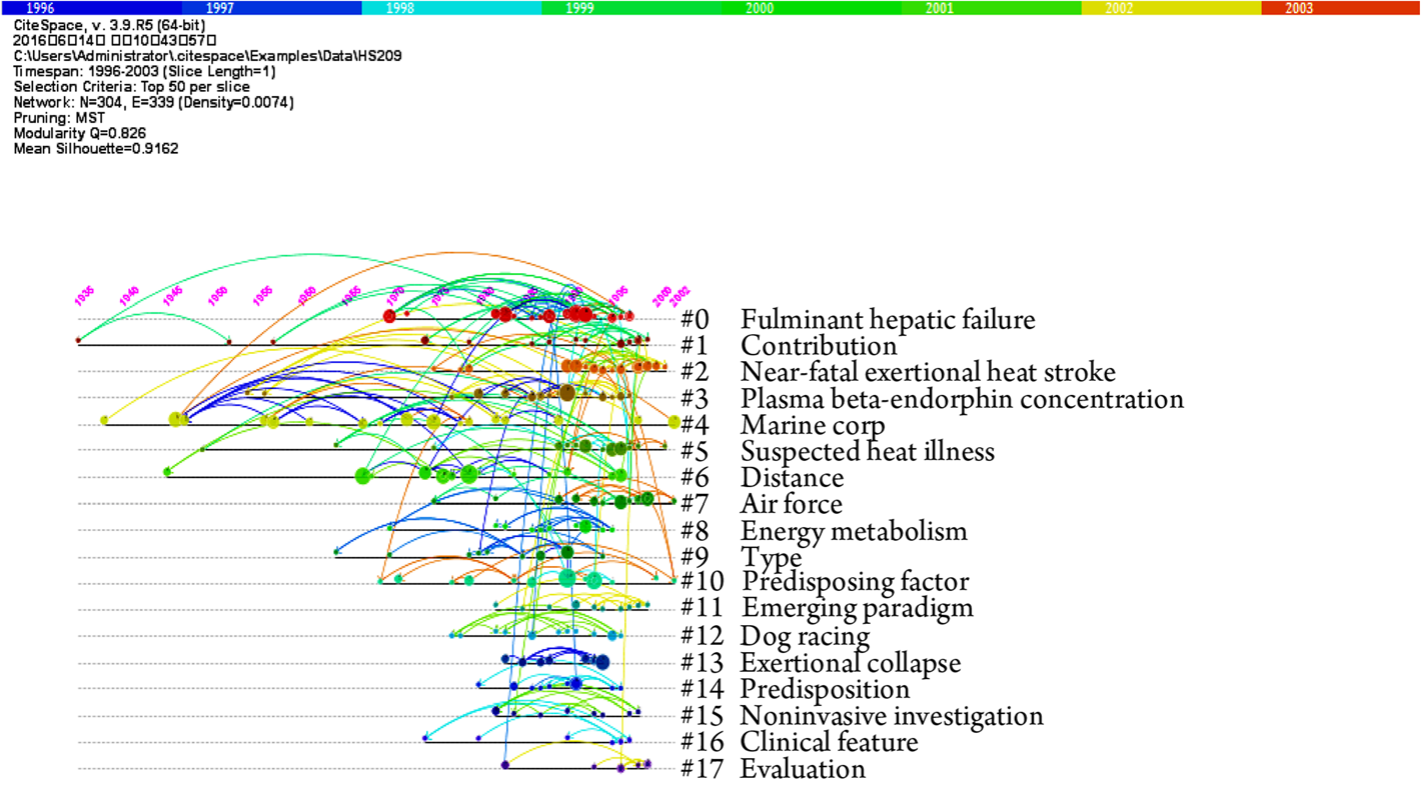
Timeline for the keyword analysis of the document co-citation clustering

The time frequency of the keywords was obtained via a co-citation analysis using CiteSpace (Fig. 2). The core documents co-cited were subject to a cluster analysis (Table 5). Fifteen categories were generated in the cluster analysis, with the following nine major categories: “fulminant hepatic failure”, “contribution”, “near-fatal exertional heat stroke”, “plasma beta-endorphin concentration”, “marine corp”, “suspected heat illness”, “distance”, “air force”, and “energy metabolism” et al. In this figure, the timeline of clusters labeled using keywords is shown horizontally. The earliest concern was “marine corp”.

**Table 5:** Documents at the nodes of the co-citation clusters

Frequency (cited times)	Centrality (article’s degree of centralized in the cluster)	Year	Co-cited articles
84	0.30	2002	Bouchama A, 2002, New Engl J Med, V346, P1978, DOI 10.1056/NEJMRA011089
51	0.06	2007	Armstrong LE, 2007, Med Sci Sport Exer, V39, P556, DOI 10.1249/MSS.0B013E31802FA199
35	0.31	1990	Costrini A, 1990, Med Sci Sport Exer, V22, P15
30	0.06	2002	Binkley HM, 2002, J Athl Train, V37, P329
29	0.17	1990	Shapiro Y, 1990, Med Sci Sport Exer, V22, P6
28	0.09	1999	Epstein Y, 1999, Med Sci Sport Exer, V31, P224, DOI 10.1097/00005768-199902000-00004
27	0.10	2007	Casa DJ, 2007, Exerc Sport Sci Rev, V35, P141
24	0.07	2005	Casa DJ, 2005, Curr Sport Med Rep, V4, P309
23	0.04	2004	Rav-acha M, 2004, Am J Med Sci, V328, P84, DOI 10.1097/00000441-200408000-00003
22	0.28	1996	Armstrong LE, 1996, Am J Emerg Med, V14, P355, DOI 10.1016/S0735-6757(96)90048-0
22	0.17	1967	Shibolet S, 1967, Q J Med, V36, P525
22	0.11	2003	Proulx CI, 2003, J Appl Physiol, V94, P1317, DOI 10.1152/JAPPLPHYSIOL.00541.2002
22	0.09	1998	Dematte JE, 1998, Ann Intern Med, V129, P173
21	0.13	2005	Smith JE, 2005, Brit J Sport Med, V39, P503, DOI 10.1136/BJSM.2004.013466
84	0.30	2002	Bouchama A, 2002, New Engl J Med, V346, P1978, DOI 10.1056/NEJMRA011089

## Discussion

According to classical bibliometric theory, increases or decreases in the number of scientific research publications indicate the speed of scientific/technological development. The present study shows that the number of published research articles on exertional heat stroke between 1996 and 2015. These publications indicate several findings. First, heat stroke research has been of continuous concern. Second, environmental heat damage, sports heat damage, and military action training heat damage remain problems that cannot be ignored. Finally, many problems have yet to be solved regarding the diagnosis, treatment, and prognosis of heat stroke.

With regard to distribution by country/region, US and Israel are the two leading countries. Mainland China published the most articles on heat stroke ranking No.4. Previous bibliometric studies in other fields have also found a sharp increase in the number of research articles from Mainland China, exceeding Hong Kong and Taiwan [11–13] and ranking second only to the US [14]. The continuous research progress in the number of published articles on exertional heat stroke demonstrates the overall improvements in critical care medicine, sports medicine, and military medicine in the output country.

With respect to journal distribution, the top 10 journals publishing articles on exertional heat stroke were all specialist publications, and none were comprehensive. On one hand, this evidence indicates that exertional heat stroke research is relatively esoteric. Most of these top 10 journals were classified as quartile 2 or 3 SCI publications. These journals mainly focus on Sport Science or Military Medicine. Although impact factor has been extensively used to evaluate the quality of research published [15], its value has always been questioned. Professor Alberts, the editor-in-chief of *Science*, the top journal in the sciences, recently published an editorial stating that impact factor has led to abnormalities in the research evaluation system [16]. Moreover, high impact factor journals occasionally publish low-quality research. Therefore, we did not analyze or discuss impact factor in the present study.

Recently, an increasing number of bibliometric analyses have emerged in medicine [11–13, 17–20] and have demonstrated significant value [21]. Previous analyses have shown that the number of published articles grew rapidly in certain countries that are emerging in scientific research, which reduced the share of articles from traditional research powers in Europe and the US [22]. A 2010 study showed that 100 classic publications in Bone Science were primarily from the UK and the US [23]. The authors of that study predicted that China would reverse this situation and establish a new balance in the near future [21]. As the present study revealed, this trend has begun to show in heat stroke research. Furthermore, we performed a visualization cluster analysis of co-cited documents on heat stroke and listed core documents in these clusters.

The present study has a few limitations. For example, to perform the citation analysis, we only searched the SCI database and not the Medline or Embase databases. However, we are certain that the SCI database generally includes all mainstream documents in the natural sciences.

## Conclusions

In summary, the research evidence gained continous attention in exertional heat stroke-related fields. USA is the dominated country in this field.

## Abbreviation

SCI: Science Citation Index
